# Reducing educational inequalities in mortality: a comparative risk assessment approach

**DOI:** 10.1093/eurpub/ckac129.402

**Published:** 2022-10-25

**Authors:** TA Eikemo

**Affiliations:** Centre for Global Health Inequalities Research, Norwegian University of Science and Technology, Trondheim, Norway

## Abstract

Educational inequalities in mortality are increasingly recognized as one of the main challenges for health policy. Studies comparing European countries have shown that such inequalities are substantial almost everywhere, but that there are important variations between countries, suggesting great scope for reduction. However, identifying this scope is difficult because it requires comparative information about the educational distribution of mortality rates, risk factors and relative risks. In this presentation I show how this can be done, by quantifying the impact of a theoretical equalization of the distribution of several known risk factors for mortality, in a comparative risk assessment approach. Harmonized data set on mortality (from register data) and risk factors (from survey data) by educational level for 21 European populations in the early 2000s were applied. The impact of the risk factors on mortality in each educational group was determined using Population Attributable Fractions (PAF). The impact on inequalities in mortality was estimated applying two counterfactual scenarios: a theoretical upward levelling scenario in which it is assumed that inequalities in the risk factor were completely eliminated, and a more realistic best practice scenario, in which it is assumed that inequalities in a risk factor were to be reduced to those seen in the country with the smallest inequalities for that risk factor. The analysis shows how information on risk factors, mortality rates and relative risks can be combined from different data sources and provide a meaningful analysis of the European mortality burden that can be linked to educational inequalities in risk factors. The analysis also shows that upward levelling scenarios and best practice scenarios demonstrate a theoretical potential for reducing inequalities in mortality.

